# Automated measurement of ascorbic acid levels in camu-camu using Vision Transformer

**DOI:** 10.3389/fpls.2025.1540535

**Published:** 2025-11-26

**Authors:** Juan Carlos Gutiérrez Cáceres, Paolo Cesar Leonel Blancas Lopez, Richard Esmith Gaviria Huanio, Nilton Cesar Ayra Apac, Euclides Panduro Padilla, Cesar Augusto Agurto Cherre

**Affiliations:** 1 Faculty Computer Science, Universidad Nacional de San Agustin, Arequipa, Peru; 2 Faculty of Engineer System, Universidad Nacional de Ucayali, Pucallpa, Peru

**Keywords:** ascorbic acid, fruit detection, RT-DETR, Vision Transformer, camu-camu

## Abstract

This study introduces a fully automated and cost effective approach to quantify ascorbic acid levels in camu-camu (Myrciaria dubia), a tropical super fruit renowned for its exceptionally high vitamin C content. Conventional analytical techniques depend on specialized laboratory equipment, limiting their applicability in field settings and among small-scale producers. To address this issue, we developed an integrated pipeline that combines a real-time Detection Transformer (RT-DETR) for precise fruit detection with a Vision Transformer (ViT) to classify fruits across four ripening stages. Building on these outputs, we designed an image-based estimation model that predicts ascorbic acid concentration using fruit size and ripening stage as key indicators. The RT-DETR achieved excellent detection performance, with a precision of 0.970 and a recall of 0.976, outperforming YOLOv8 (0.946 and 0.913, respectively). Likewise, the ViT classifier reached a precision of 0.970, surpassing VGG16, which achieved 0.946. The proposed estimation model yielded a very low prediction error, confirming its reliability. Overall, this work offers a practical, scalable, and accurate solution for estimating ascorbic acid directly from images, delivering significant benefits to producers and advancing the application of computer vision in the pharmaceutical industry.

## 1 Introduction

Camu-camu (Myrciaria dubia) is a tropical super fruit widely valued for its extraordinary ascorbic acid content, reaching up to 2780 mg per 100g of fresh fruit. This exceptionally high vitamin C concentration drives its commercial value, as prices are closely tied to ascorbic acid levels in harvested fruit. For farmers, accurate quantification of this compound is essential, as it directly influences income and market competitiveness. However, current field assessment remains challenging because available techniques rely on laboratory-based physicochemical analyses, which are expensive, time-consuming, and inaccessible for most small-scale producers.

In Peru, the National Institute of Quality (INACAL) established the technical standard NTP-NA 0085:2011 (revised in 2021), which outlines classification criteria and minimum quality requirements for the commercialization of fresh camu-camu (INACAL, 2017). While this standard provides a regulatory framework, it does not eliminate the dependence on laboratory testing, leaving producers without a practical, on-site solution for quality evaluation.

To overcome this limitation, this study proposes an automated approach for estimating ascorbic acid levels directly from images using advanced computer vision techniques. The proposed pipeline integrates a real-time Detection Transformer (RT-DETR) for accurate fruit localization with a Vision Transformer (ViT) to classify fruits into four ripening stages. By leveraging visual attributes specifically size and ripening stage to estimate ascorbic acid concentration, the system delivers accurate predictions without the need for specialized equipment. This method reduces costs, improves assessment efficiency, and makes quality evaluation accessible to producers.

Traditional methods for ascorbic acid measurement, such as iodometric titration and colorimetric assays, typically require specialized laboratory equipment, including high-precision analytical balances, spectrophotometers (e.g., UV-Vis Spectrophotometer), burettes, and colorimeters. These instruments, while precise, are labor-intensive, expensive, and impractical for rapid field analyses. Recent advances in computer vision have enabled automated approaches for ascorbic acid estimation. However, traditional methods remain the gold standard due to their high accuracy, despite being time-consuming and resource-intensive.

The remainder of this article is organized as follows. Section 2 reviews related literature; Section 3 describes the proposed methodology for fruit detection and ripening stage classification; Section 4 presents the experimental results; and Section 5 discusses the conclusions and outlines potential directions for future work.

## 2 Literature review

Deep learning has been widely explored for classifying fruits based on ripeness and quality. For instance (Al Haque et al., 2021), developed a convolutional neural network (CNN) that achieved 93.4% precision in identifying banana ripeness. Similarly (Li et al., 2021), combined a five-layer CNN with Random Forest and K-Nearest Neighbors (KNN) to classify various fruits including bananas, apples, strawberries, oranges, and mangoes with high accuracy.

In related work (Iqbal and Hakim, 2022), evaluated eight mango varieties using VGG16, ResNet152, and Inception v3, with Inception v3 achieving 99.2% precision. For strawberries (Ni et al., 2021), reported 95.75% accuracy by applying AlexNet with data augmentation techniques on images collected both in laboratory and field conditions. Low-cost solutions have also been proposed for tomatoes (Das et al., 2021): achieved 100% accuracy in classifying ripeness, while (Hsieh et al., 2021) used an RCNN to identify ripe tomatoes with accuracy exceeding 95%.

Although the use of transformers in computer vision remains relatively new, these models have shown remarkable improvements over traditional CNNs, particularly in capturing complex feature relationships. For example (Chen et al., 2022), applied YOLOv5 to detect citrus fruits and ResNet34 to classify ripeness, achieving 95.07% accuracy. Similarly (Qiu et al., 2022), enhanced YOLOv4 with SM-YOLOv4, obtaining an average accuracy of 93.52% for mango ripeness detection. Likewise (Zhang et al., 2022), proposed an improved YOLOv5 incorporating GhostNet and CBAM to detect the ripeness of Hemerocallis citrina Baroni, reaching an accuracy of 84.9%.

In the case of camu-camu, however, research on automated determination of ascorbic acid remains scarce. Most existing studies have focused on laboratory-based assessments of ripeness and vitamin C content. For example (Icumina, 2005), reported that the highest ascorbic acid levels occur at the ripe pinton stage, while (Pablo et al., 2020) used ultrasound to examine how ripening affects bioactive components. Although valuable, these studies do not address automation or the use of machine vision techniques, leaving a clear research gap that our work aims to fill.

Recent advances in computer vision have demonstrated the superior ability of transformer-based architectures, such as RT-DETR and Vision Transformer (ViT), to capture complex spatial relationships and detailed visual features, surpassing traditional CNN-based models like YOLO or Faster R-CNN. These models excel in challenging scenarios, including densely packed and overlapping objects, which are common in Camu-Camu fruit images. Furthermore, advanced image enhancement techniques, such as super-resolution with GANs (Cun et al., 2019), and highlight removal with Attentive GANs (Ledig et al., 2017), have significantly improved the clarity and reliability of image-based analyses. Meta-learning approaches, as described in ()?, offer additional improvements in image resolution and analysis accuracy.

Recent advances in image-based nutrient assessment also warrant attention. Wang et al. (2021) and Zhang et al. (2023) demonstrated the effectiveness of multispectral and hyperspectral imaging for estimating nutrient content through analysis of fruit spectral and dimensional features. These methods provide non-destructive, high through put alternatives to traditional techniques. Similarly, Martinez et al. (2022) explored the use of 3D imaging to evaluate fruit quality attributes, including vitamin C levels, highlighting the potential of combining dimensional and spectral data to enhance prediction accuracy.

Building on these developments, our research introduces the application of transformers for camu-camu detection and classification while also presenting an innovative approach that visually correlates fruit size and ripeness with ascorbic acid levels. This method achieves an average estimation error of only 7% and represents a significant contribution to the use of machine vision in agriculture, improving both accuracy and accessibility for producers.

Furthermore, combining laboratory analyses with computer vision techniques opens the door to a promising hybrid approach. Laboratory methods remain essential for accurately measuring compounds such as ascorbic acid, while vision-based tools allow for rapid, non-invasive, and scalable evaluations directly in the field. Together, these approaches complement each other, offering a practical pathway to improve accuracy, expand applicability, and foster the adoption of these technologies in real agricultural settings.

## 3 Materials and methods

**Figure 1:**
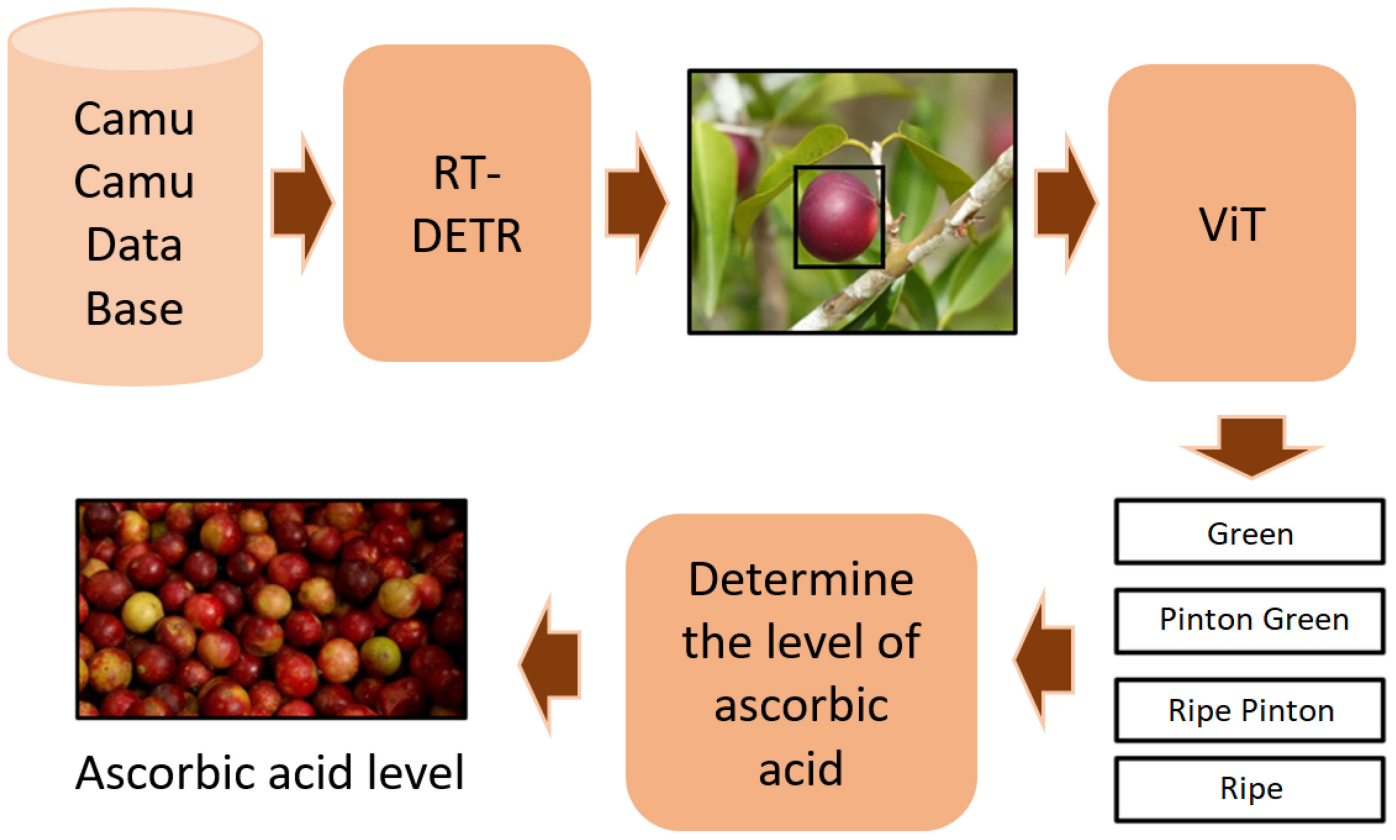
Pipeline of the proposed framework.

**Figure 1** illustrates the post-harvest image analysis pipeline designed for camu-camu fruits. High-resolution images were captured using an iPhone 13 Pro camera. These images were first processed by the RT-DETR model, which excels at detecting and isolating individual camu-camu fruits in each frame. RT-DETR, built on an enhanced version of the DEtection TRansformer (DETR), employs attention mechanisms to accurately locate and segment fruits within the images. Once detected, the segmented fruits were classified using a Vision Transformer (ViT) model. The ViT leverages transformer-based architectures to recognize complex visual patterns, assigning each fruit to one of four maturity stages as defined by the Peruvian technical standard: Green, Pinton Green, Ripe Pinton, and Ripe. This two-step approach detection followed by classification forms the foundation of our automated pipeline.

The reason using RT-DETR and Vision Transformer (ViT) models were specifically chosen due to their superior ability to capture complex spatial relationships and detailed visual features compared to conventional CNN-based methods like YOLO or Faster R-CNN. RT-DETR integrates transformer architectures directly into object detection, improving performance on densely packed and overlapping fruits common in Camu-Camu images. The ViT model provides robust classification through its self- attention mechanisms, excelling in recognizing subtle visual differences linked to varying ripeness stages, surpassing traditional convolution-based models.

**Figure 2:**
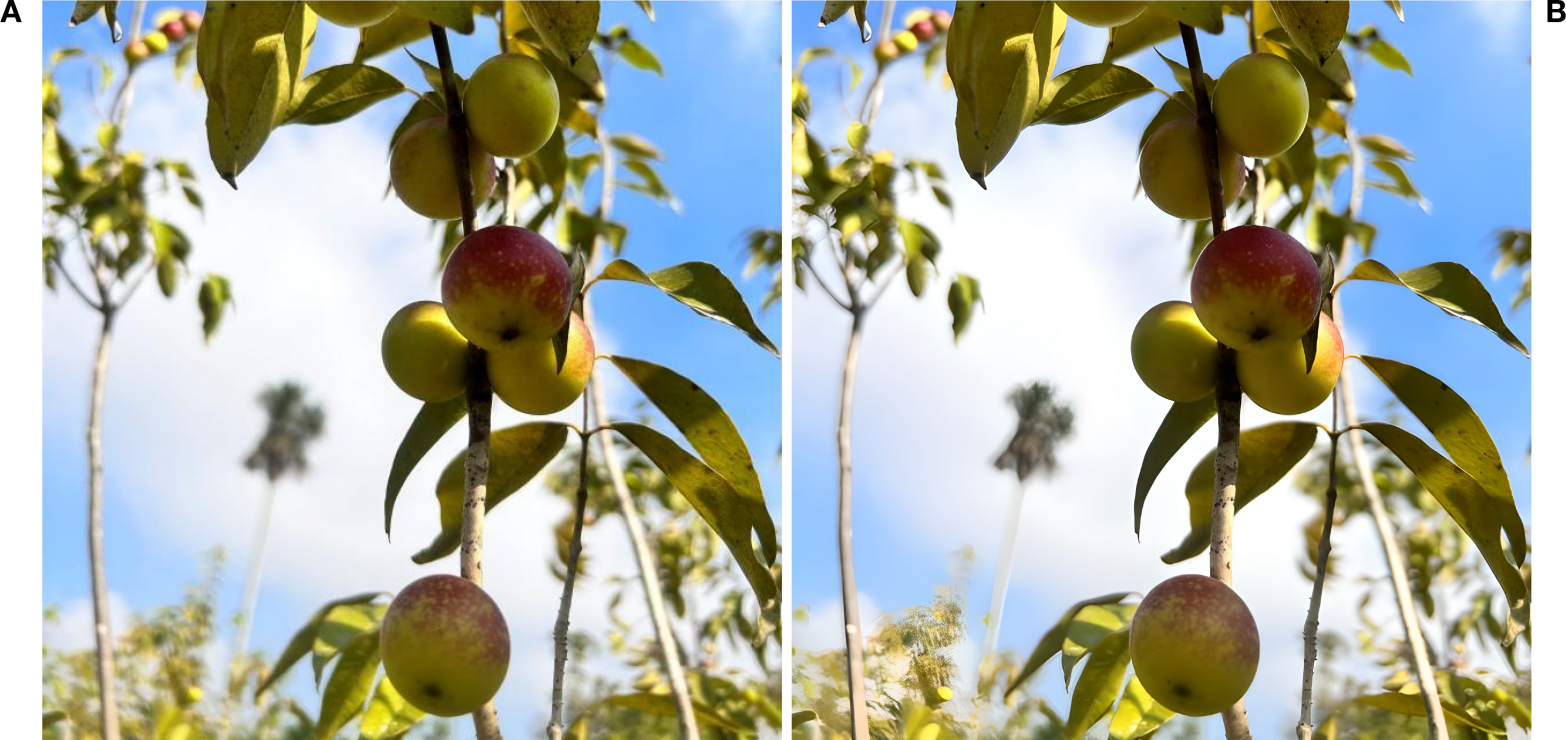
Comparison of image resolution and detection. **(A)** Original image. **(B)** Super-resolved image using GANs, showing enhanced clarity and detail for Camu-Camu fruits.

To enhance the clarity and precision of Camu-Camu fruit images, we propose leveraging advanced super-resolution techniques. For instance, methods demonstrated by *Scientific Reports*, such as retinal fundus image super-resolution using generative adversarial networks (GANs), **Figure 2**.

High-resolution images of Camu-Camu fruits were first enhanced using Real-ESRGAN, a GAN-based super-resolution algorithm, to improve image clarity. Object detection was performed using RT-DETR, while fruit classification was conducted using a Vision Transformer (ViT) model. To reduce glare and reflections, we applied an Attentive GAN-based highlight removal method prior to analysis.

### 3.1 Hardware requirements

**Figure 3:**
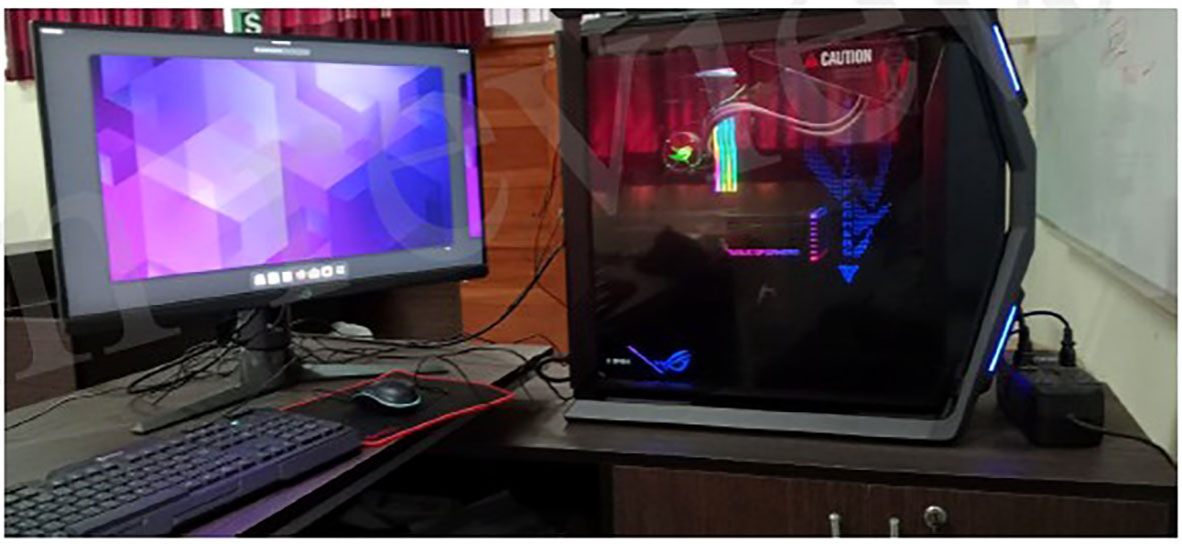
Hardware – server.

The experiments were conducted on a workstation equipped with an NVIDIA RTX 5060 GPU (24 GB VRAM), an Intel i9 processor, and 32 GB RAM. in the **Figure 3** This configuration allowed real-time fruit detection, classification, and ascorbic acid estimation. The system is scalable to less powerful hardware with longer processing times.

### 3.2 Creation of the camu-camu image database

The image database was developed as part of a research project at the University of Ucayali. Fieldwork involved collecting camu-camu fruits at different ripening stages directly from plantations.

Images were captured under controlled conditions with an iPhone 13 Pro (12 MP), producing photographs at 3024 × 3024 pixels. Uniform lighting was ensured using a 5000K white light source, and images were taken between 10:00 AM and 3:00 PM to maximize natural light. Fruits were placed on a neutral-colored background to enhance contrast and simplify detection.

**Figure 4:**
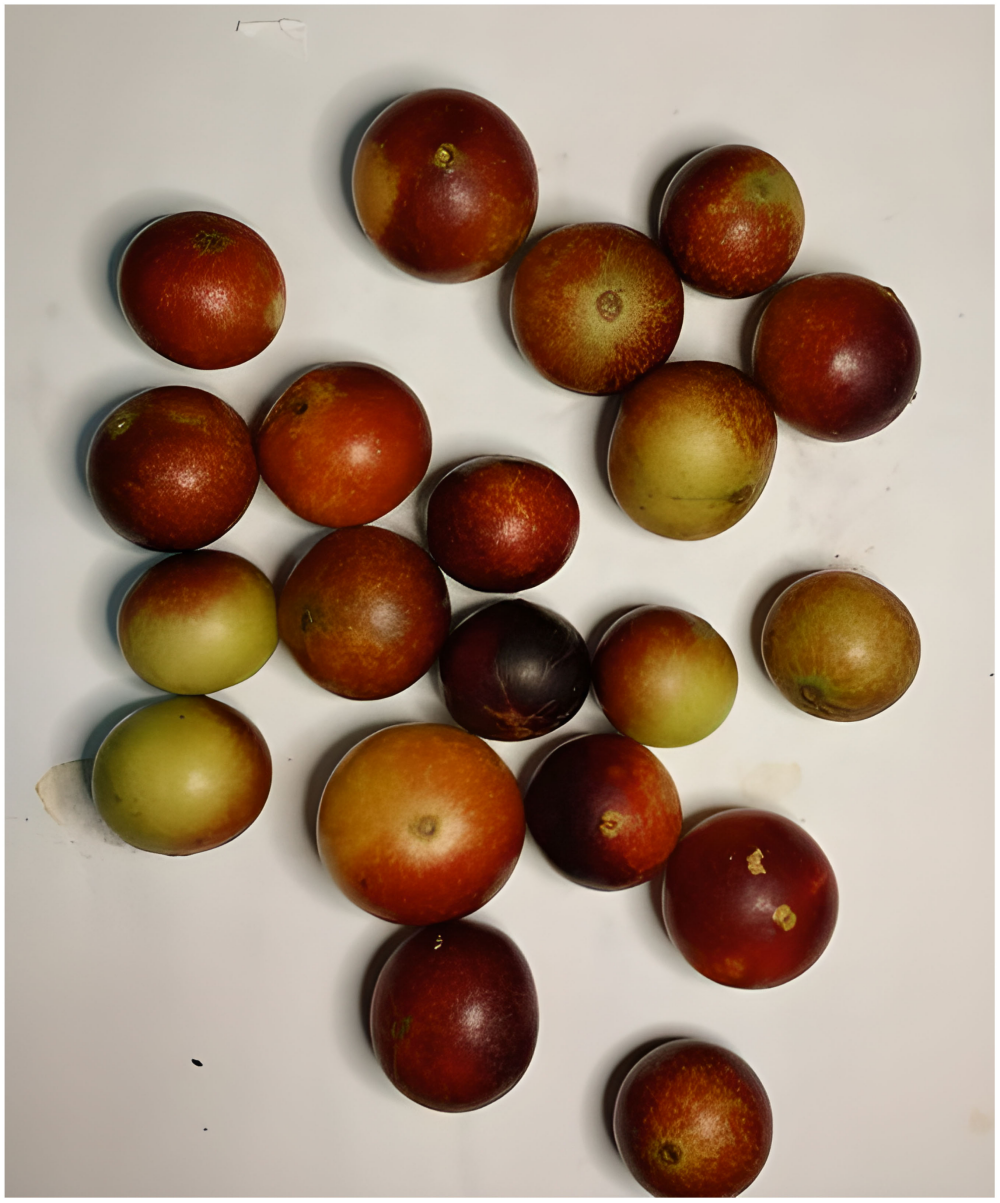
Example of a camu-camu image from the database.

A total of 1592 images were collected, each containing approximately 15 fruits at varying ripeness stages (Green, Pinton Green, Ripe Pinton, and Ripe). The camera was positioned 30 cm above the samples at a fixed angle of 90°, ensuring consistency across all captures. Images were split into training (60%), validation (20%), and test (20%) sets. **Figure 4** shows an example of an image from the dataset.

### 3.3 Fruit detection using a transformer

Images for detection are captured directly through a dedicated mobile application, which serves as the primary user interface for data acquisition in real-world scenarios. Once an image is taken by the user, it is automatically transmitted to a remote server running a Flask-based backend. All subsequent processing—including fruit detection using RT-DETR, classification, and ascorbic acid estimation—is performed server-side, leveraging the computational power of the cloud infrastructure. This client-server architecture enables real-time analysis and results delivery, ensuring a seamless and scalable workflow suitable for both field and laboratory environments.

RT-DETR combines the strengths of Vision Transformers with a hybrid encoder that efficiently processes multi-scale features by separating intra-scale interactions and merging information across scales. This design enhances both accuracy and real-time detection capabilities while reducing computational costs.

YOLOv8, although a strong baseline, demonstrated lower accuracy in detecting camu-camu. Various model sizes (n, s, m, l, x) were tested, with the medium-sized version providing the best balance between speed and accuracy. Larger versions offered no significant performance gain and were computationally more demanding.

**Figure 5:**
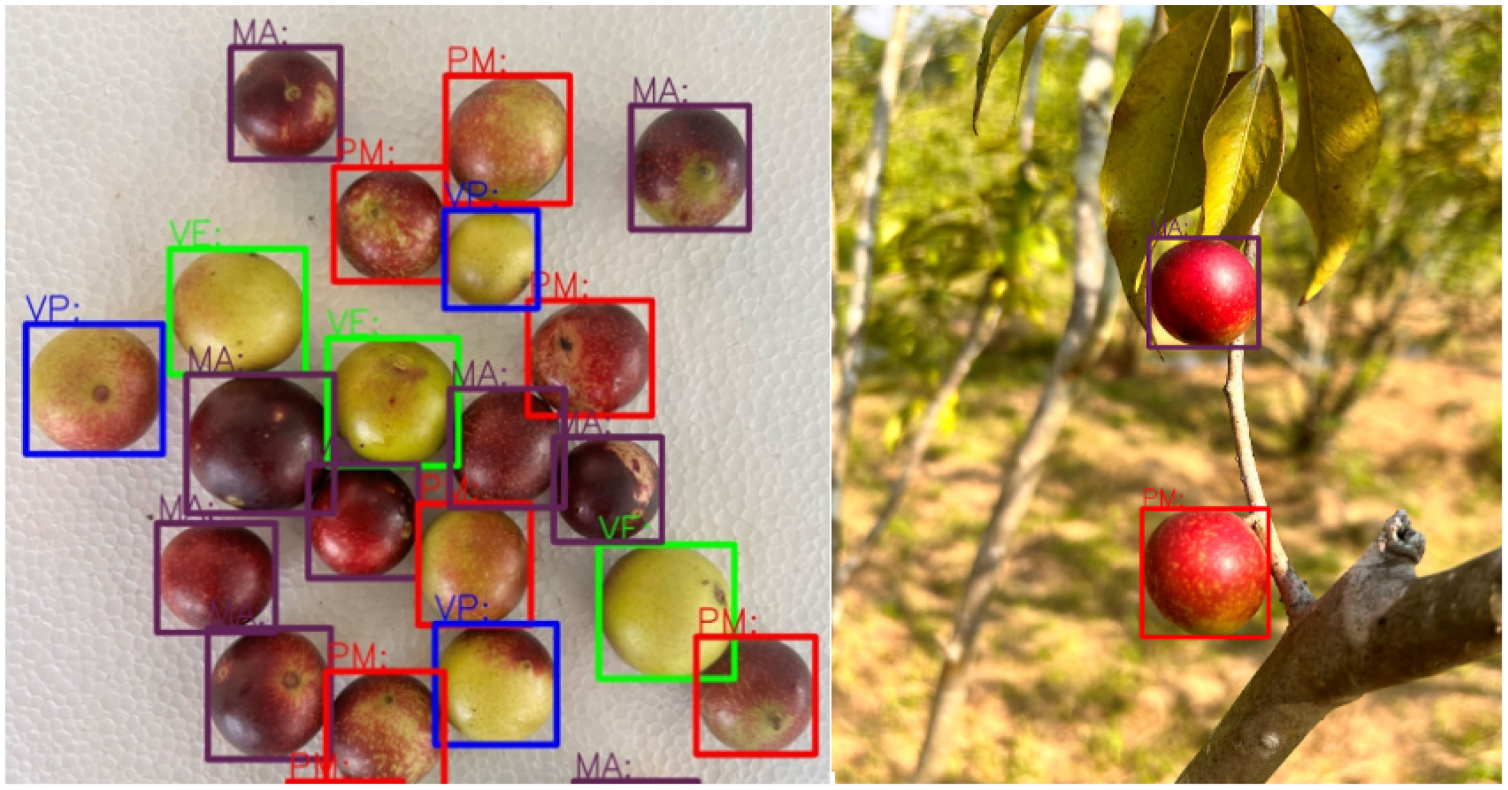
Detection results under various backgrounds.

**Figure 5** shows detection examples in both controlled and farm environments, illustrating RT-DETR’s robustness across different conditions. **Table 1** details the dataset split for detection.

**Table 1:** Details of the detection dataset.

Detection dataset	
Set type	Number of images
Training	956 (60%)
Validation	318 (20%)
Test	318 (20%)

The model was pre trained on COCO (Lin et al., 2015) and used ResNet-50 for feature extraction. Training employed AdamW optimization with an initial learning rate of 2×10^−4^, weight decay of 0.1, batch size of 16, and 100 epochs. Data augmentation techniques random flips, cropping, and scaling were applied to improve generalization.

### 3.4 Classification according to maturity stage

For fruit classification, a Vision Transformer (ViT) architecture was implemented to discriminate between the four ripening stages of camu-camu. The dataset was partitioned into training (60%), validation (20%), and test (20%) subsets. Ripeness labels were assigned and cross-validated by four expert farmers with extensive experience in camu-camu production, ensuring compliance with the Peruvian classification standard.

**Table 2** presents the detailed distribution of images across the training, validation, and test sets, including the number of samples per ripening stage.

**Table 2:** Distribution of images in the classification dataset.

Classification dataset	
Set type	Number of images
Training set	3827 (60%)
Green	723
Pinton Green	766
Ripe Pinton	715
Ripe	1623
Validation set	1275 (20%)
Green	255
Pinton Green	241
Ripe Pinton	238
Ripe	541
Test set	1277 (20%)
Green	255
Pinton Green	241
Ripe Pinton	239
Ripe	542

**Figure 6:**
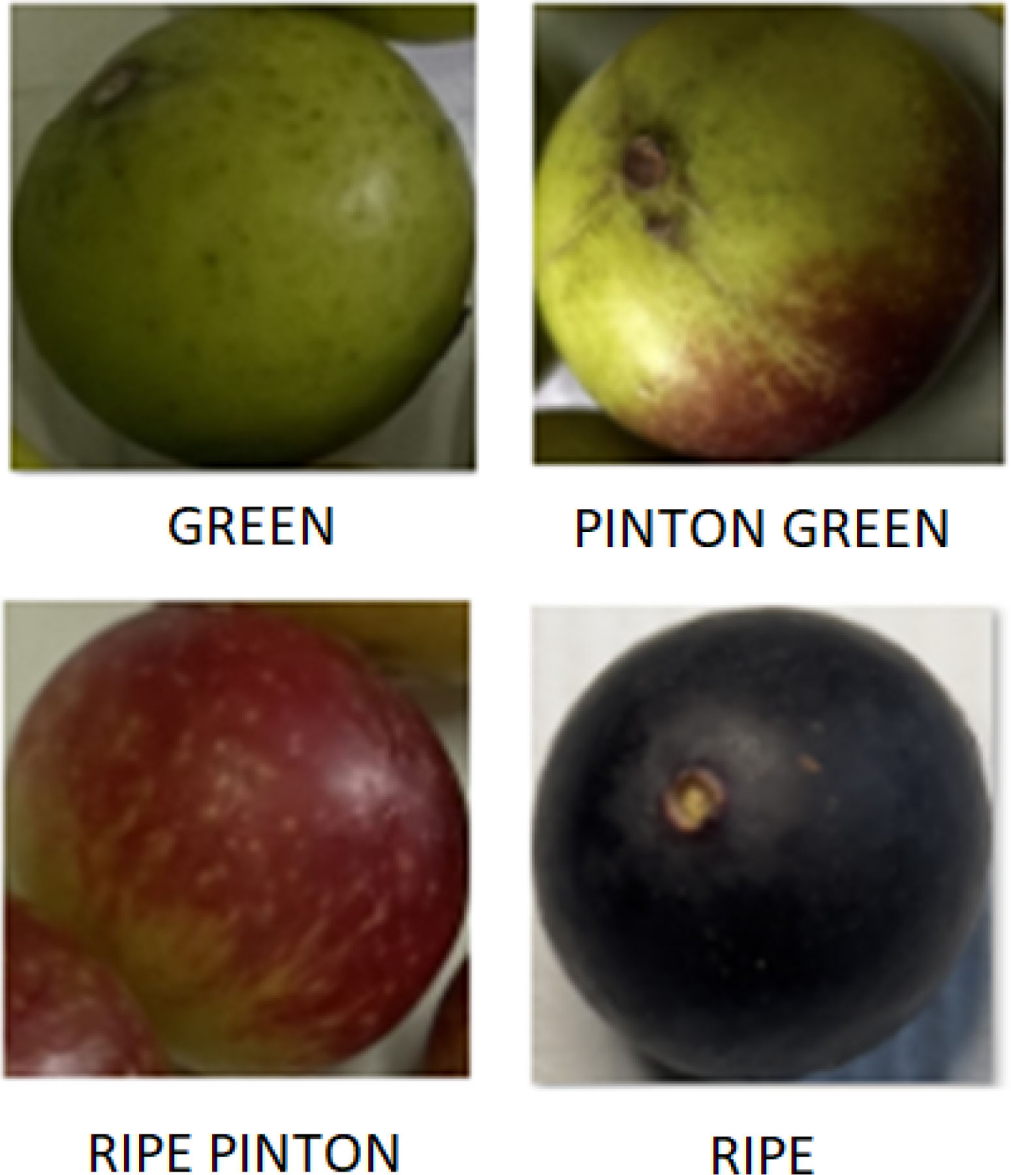
Representative images of the four ripening stages: Green, Pinton Green, Ripe Pinton, and Ripe.

Examples of the four ripening stages green, Pinton Green, Ripe Pinton, and ripe are shown in **Figure 6**. These samples illustrate the visual differences used as the basis for classification.

The ViT model processed images resized to 72 × 72 pixels and divided them into 6 × 6 patches. The architecture consisted of eight transformer blocks, each with four multi-head attention heads, and a final classification layer producing four output classes. Training was performed using the AdamW optimizer (learning rate 0.001, weight decay 0.0001), sparse categorical cross entropy loss, a batch size of 256, and 100 epochs. Checkpointing was applied to retain the best performing model based on validation accuracy.

Data augmentation techniques including resizing, random flips, rotations, zooming, and normalization—were employed to increase dataset variability. These transformations effectively tripled the number of training samples processed per epoch, enhancing the model’s generalization capacity.

### 3.5 Determination of ascorbic acid levels

Each detected and classified fruit was first normalized by its pixel area (143.79px/cm) and converted to a physical area (cm^2^). Assuming an average pulp thickness of 8mm and a density of 1g/cm^3^, that area was converted into pulp volume and then into mass (m_pulp_). Unripe (green) fruits—reserved for subsequent ripening—were excluded from the final analysis.

For ground-truth measurements, 100 g of camu camu fruits were weighed for each ripening stage, corresponding to approximately 10 fruits per sample. The pulp was carefully separated from the seeds and skin, then diluted with 100 mL of distilled water to obtain homogenized solutions. From each stage, ten independent aliquots were prepared, allowing replicate titrations to calculate the mean concentration and associated experimental error. These solutions were analyzed using the 2,6-dichlorophenolindophenol (DCPIP) titration method following standardized laboratory protocols.

The ascorbic acid concentration (mgAA/g of pulp) obtained via DCPIP titration was multiplied by m_pulp_ to yield the total ascorbate content per fruit. Mean values and standard errors were computed from the ten replicates.

Certified reference analyses were carried out by Natura Analítica S.A.C. (an accredited testing laboratory), yielding ascorbic acid concentrations of 1539.40mgAA/100g (Green), 1790.37mgAA/100g (Turn–Green), 2033.32mgAA/100g (Turn–Ripe), and 2187.50mgAA/100g (Ripe). These certified values were used to validate our automated estimates, as summarized in **Table 3**.

**Table 3:** Comparison between laboratory measurements and system estimates.

Comparative results	
Pinton Green	Level (mg/100g)
System	1576.42
Laboratory	1790.37
Ripe Pinton	Level (mg/100g)
System	2110.55
Laboratory	2033.32
Ripe	Level (mg/100g)
System	2309.21
Laboratory	2187.50

## 4 Results

The results are presented in two parts, focusing on fruit detection and classification. For each processed image, the system automatically estimates the ascorbic acid content based on the criteria described in the previous section.

### 4.1 Detection process

We compared the detection performance of RT-DETR, YOLOv8, and YOLOX. Across all tests, RT-DETR consistently achieved superior results. YOLOX, although competitive, performed slightly below RT-DETR in accurately detecting and isolating camu-camu fruits.

The RT-DETR model was fine-tuned from weights pre-trained on the COCO dataset (Lin et al., 2015), which contains over 30,000 images. The primary hyperparameters included a base learning rate of 0.0002, backbone 200 learning rate of 0.00002, weight decay of 0.001, gradient clipping of 0.2, 2500 warm-up steps, and EMA decay of 0.9999.

**Figure 7:**
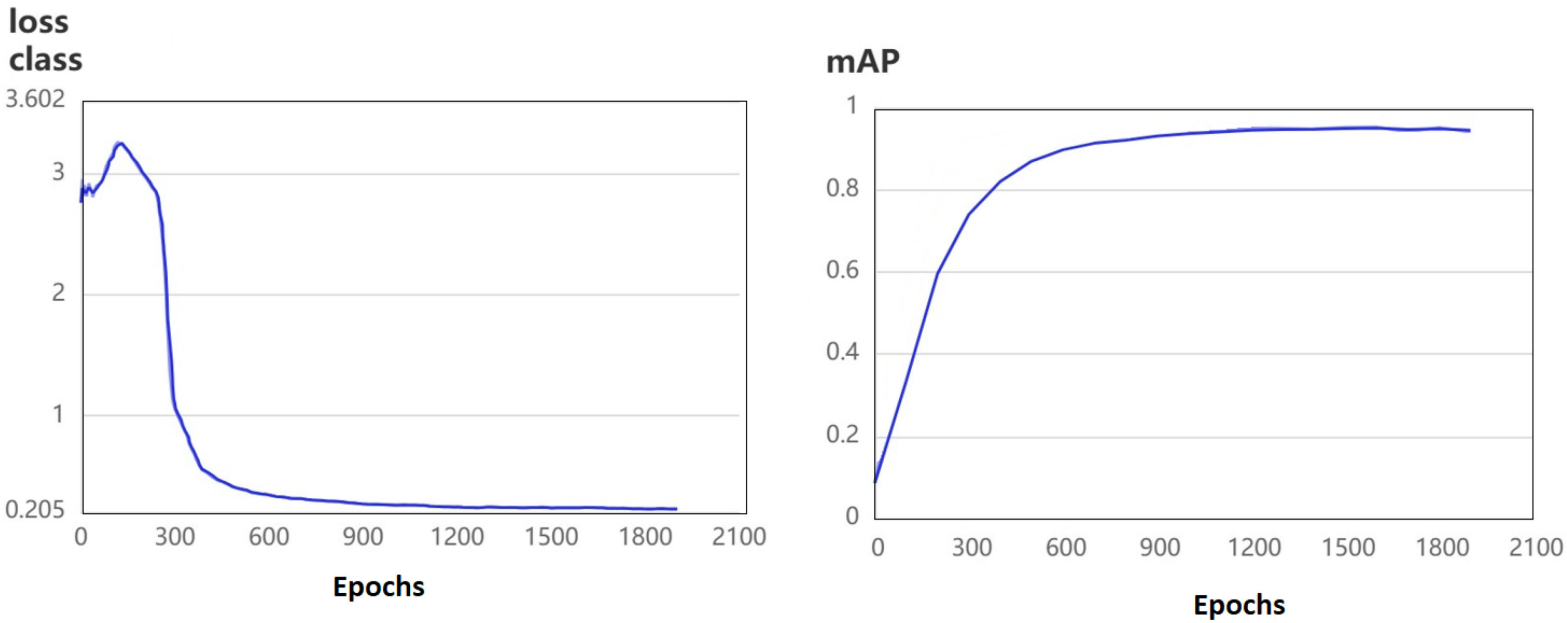
Training curves for loss and precision in the detection stage.

**Figure 7** shows the loss and precision curves during training, where convergence was observed around epoch 900.

**Table 4** summarizes the detection performance. RT-DETR achieved a precision of 0.970 and a recall of 0.976, outperforming YOLOv8 (0.946 and 0.913, respectively) and YOLOX (0.924 and 0.942). These results demonstrate RT-DETR’s superior ability to detect camu-camu fruits across various conditions.

**Table 4:** Detection performance and inference speed for different models (measured on NVIDIA RTX 5080, batch size = 128).

Detection results					
Model	Precision	Recall	mAP@50-95	F1-score	Speed (ms/img)
RT-DETR	0.970	0.976	0.973	0.931	20
YOLOv8	0.946	0.913	0.929	0.912	13
YOLOX	0.924	0.942	0.933	0.893	15

Although processing speed was not the main focus of this study, RT-DETR achieved 0.41 FLOPs, confirming its efficiency in terms of computational resources.

### 4.2 Classification process

For classification, the detected fruit images were processed using the ViT model. Hyperparameters included a learning rate of 0.002, weight decay of 0.0002, batch size of 128, 16 transformer layers, and an MLP head with 2048 and 4 units.

**Figure 8:**
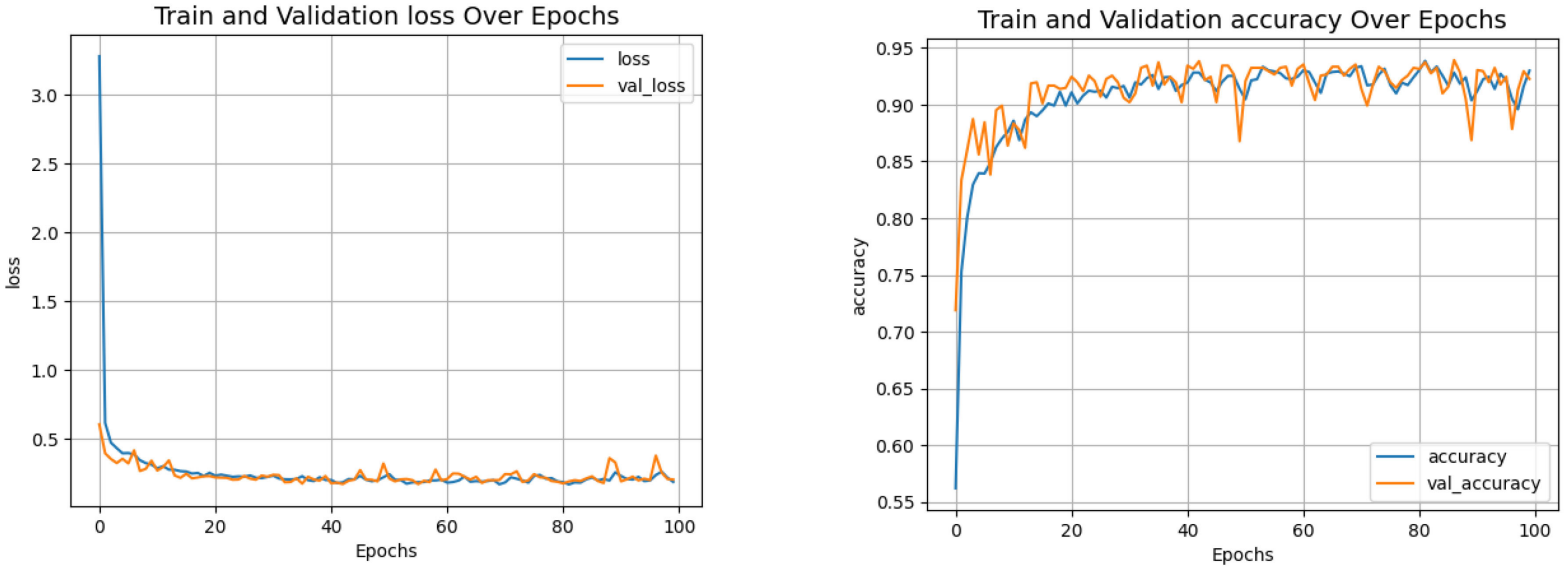
Training and validation curves classification.

**Figure 9:**
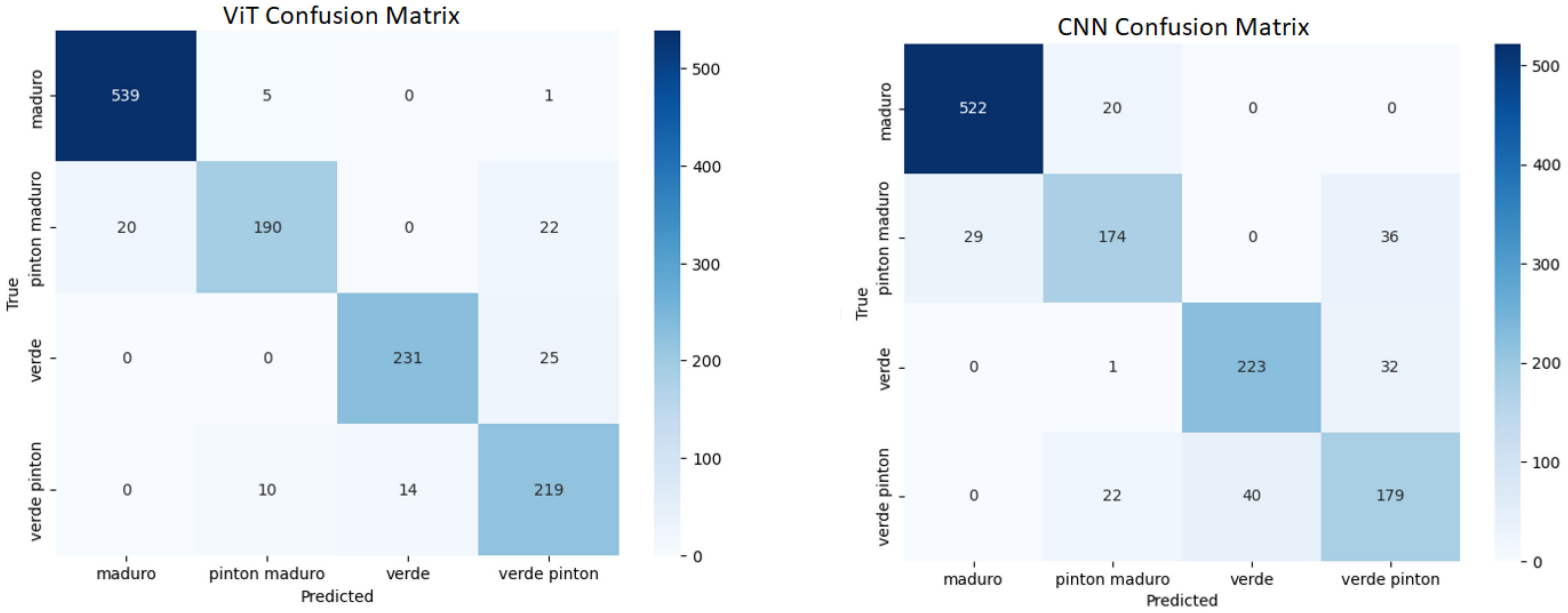
Confusion matrices for ViT and VGG16.

**Figure 8** shows the loss and accuracy curves, indicating convergence after approximately 20 epochs. **Figure 9** presents the confusion matrices comparing the ViT and VGG16 models. Although VGG16 achieved solid results, ViT consistently outperformed it.

**Table 5** shows the classification results. ViT achieved a precision of 0.970, surpassing VGG16, which reached 0.946. These results confirm the advantage of transformer-based architectures over traditional CNNs for this task.

**Table 5:** Classification performance by ripeness stage for ViT and VGG16 models.

Classification results				
Model	Accuracy	Precision	Recall	F1-score
ViT	0.970	0.972	0.971	0.971
VGG16	0.946	0.950	0.945	0.947

### 4.3 Manual depulping calibration

To establish the relationship between fruit surface area and pulp yield, a manual depulping procedure was performed on 100 samples, each consisting of approximately 10 camu camu fruits, covering all ripening stages. Unlike approaches that rely on predefined estimation models, this study directly measured the pulp content for each 100 g batch, following standardized laboratory practices previously described in the literature review.

For each sample, the projected fruit area was recorded, and the pulp was manually separated and weighed. This manual calibration approach enabled the derivation of an empirical relationship between the measured area and the corresponding pulp yield, avoiding reliance on computational estimation models (**Table 6**).

**Table 6:** Pulp yield results from manual depulping across area ranges and ripening stages.

Result pulp content			
Stage	Pulp (g)	Weight (g)	Area (cm^2^)
Ripe	12.30	100	more than 8
Ripe	8.15	100	between 5 and 8
Ripe	6.10	100	less than 5
Pinton Ripe	14.12	100	more than 8
Pinton Ripe	9.18	100	between 5 and 8
Pinton Ripe	7.20	100	less than 5
Pinton Green	10.21	100	more than 8
Pinton Green	7.33	100	between 5 and 8
Pinton Green	5.26	100	less than 5

From these measurements, an average pulp yield was computed for each area class:


$$P=0.084\;A^{2}-0.252\;A+6.292+0.63\;M$$

where:

- *P* is the estimated pulp content (g).
- *A* is the projected fruit area (cm^2^).
- *M* is the maturity factor (0, 1, or 2).

**Figure 10:**
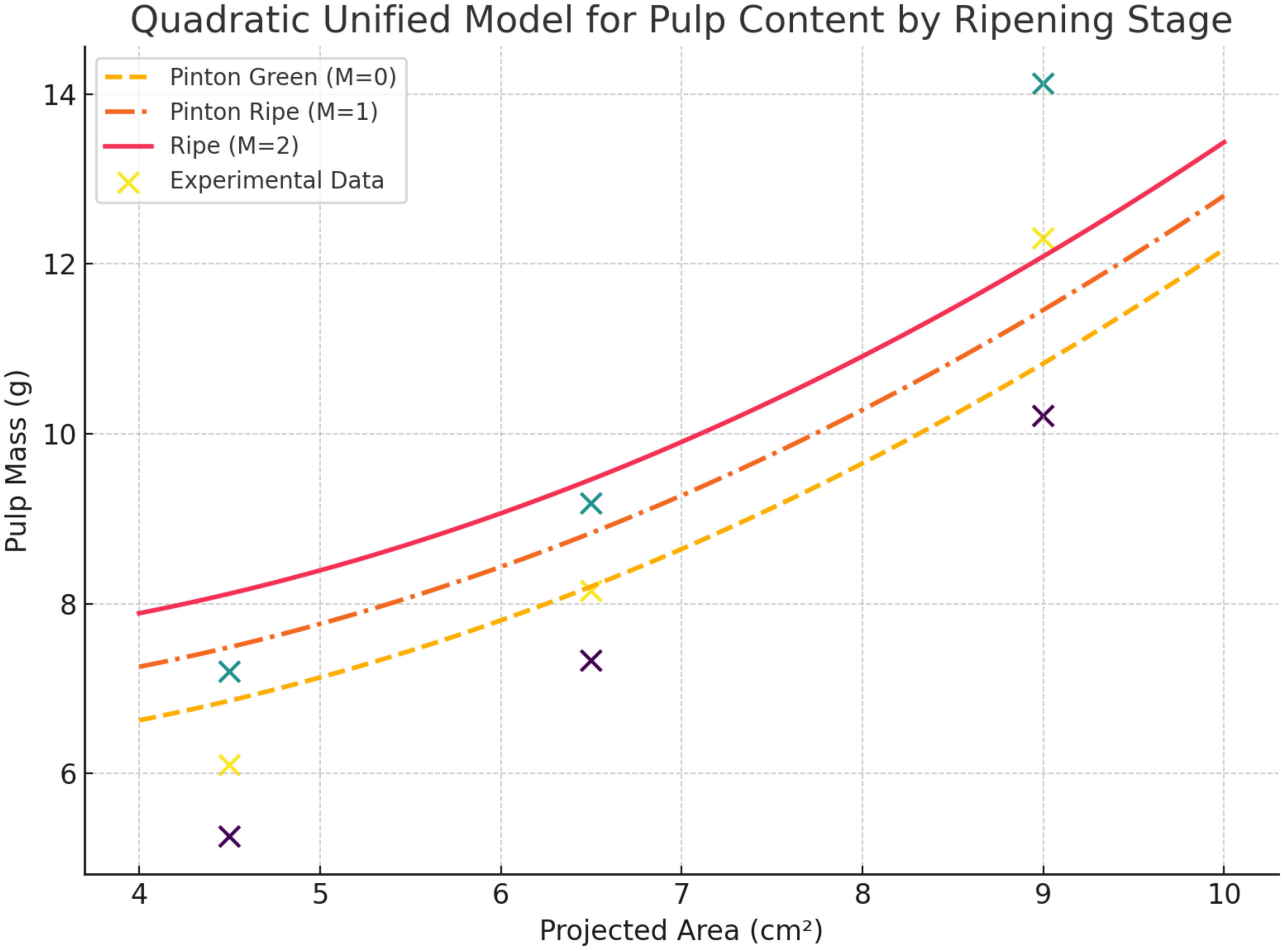
Unified quadratic model.

This calibration forms the cornerstone of the estimation pipeline, ensuring that the model accurately reflects the real-world biological relationship between fruit area, ripening stage, and pulp yield. **Figure 10** illustrates the integration of this calibration into the detection and estimation workflow.

### 4.4 Final ascorbic acid estimation

**Figure 11:**
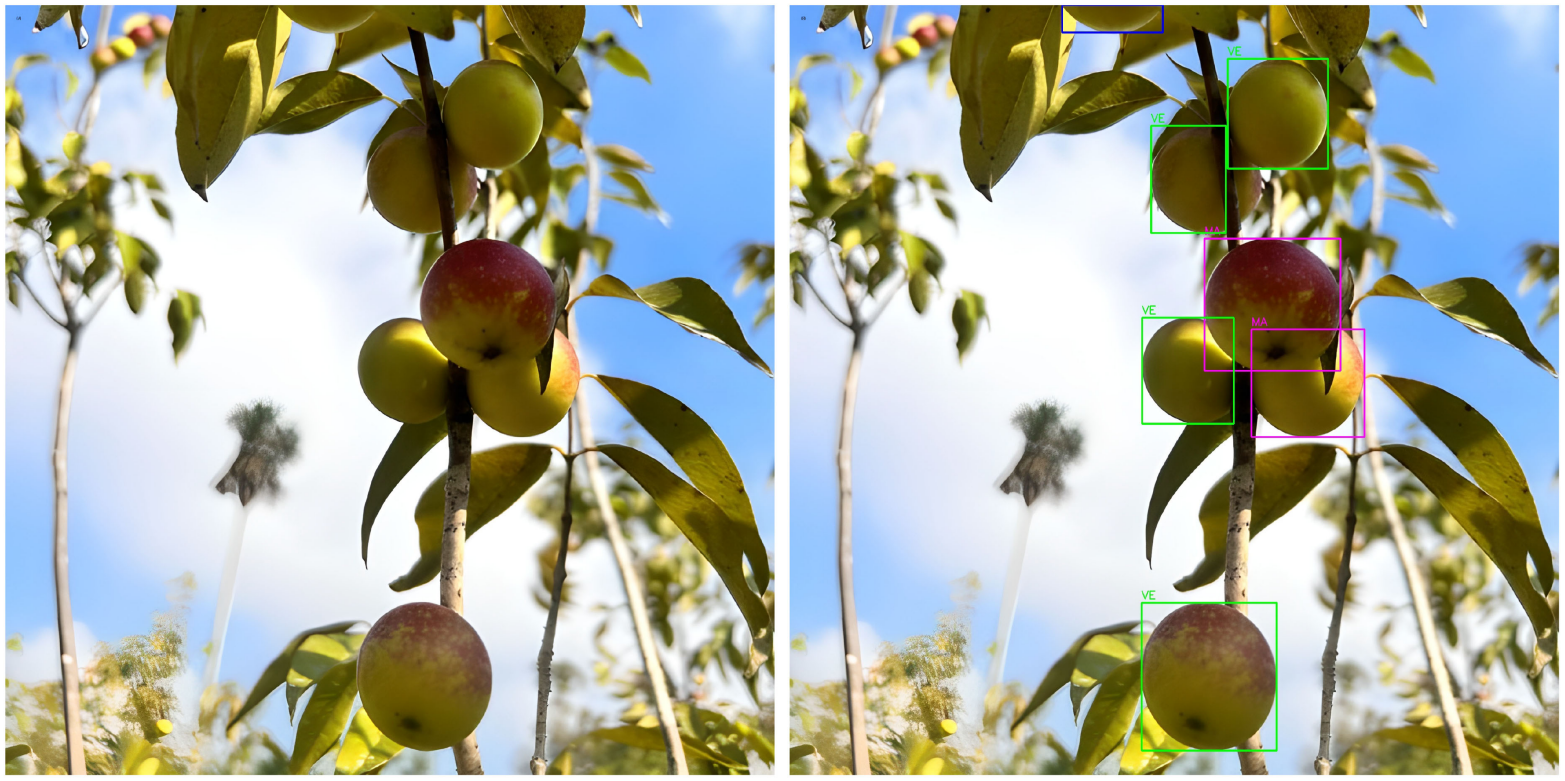
Example of detection, classification, and ascorbic acid estimation in field images.

The final stage of the pipeline integrates detection and classification to estimate ascorbic acid content for each fruit in the image. **Figure 11** illustrates this process, where fruits are labeled by ripeness stage: Ripe, Ripe Pinton, Pinton Green, and Green. The system estimates total ascorbic acid by combining detected fruit area, estimated pulp mass, and ripeness-specific vitamin C levels.

For each fruit, the system outputs measurements such as physical size, ripeness stage, and estimated pulp volume. Ascorbic acid content ( $R_{2}$) is estimated by relating laboratory values ( $R_{1}$) and reference mass ( $b_{1}$) to the mass calculated for each fruit ( $b_{2}$), using:


$$R_{2}=\frac{R_{1}\times b_{2}}{b_{1}}$$

where:

- *R*_2_ is the ascorbic acid content estimated by the proposed system (mg/100 g).
- *R*_1_ is the ascorbic acid concentration determined through laboratory titration (mg/100 g).
- *b*_1_ is the mass of the reference sample processed in the laboratory (g).
- *b*_2_ is the mass of the fruit calculated by the proposed system (g).

This proportional approach enables automated quantification of ascorbic acid without exhaustive laboratory analysis, maintaining accuracy while increasing throughput. A reference area interval of 2.5 cm^2^ was established experimentally from ten laboratory trials, providing a baseline for pulp yield estimation.

**Figure 12:**
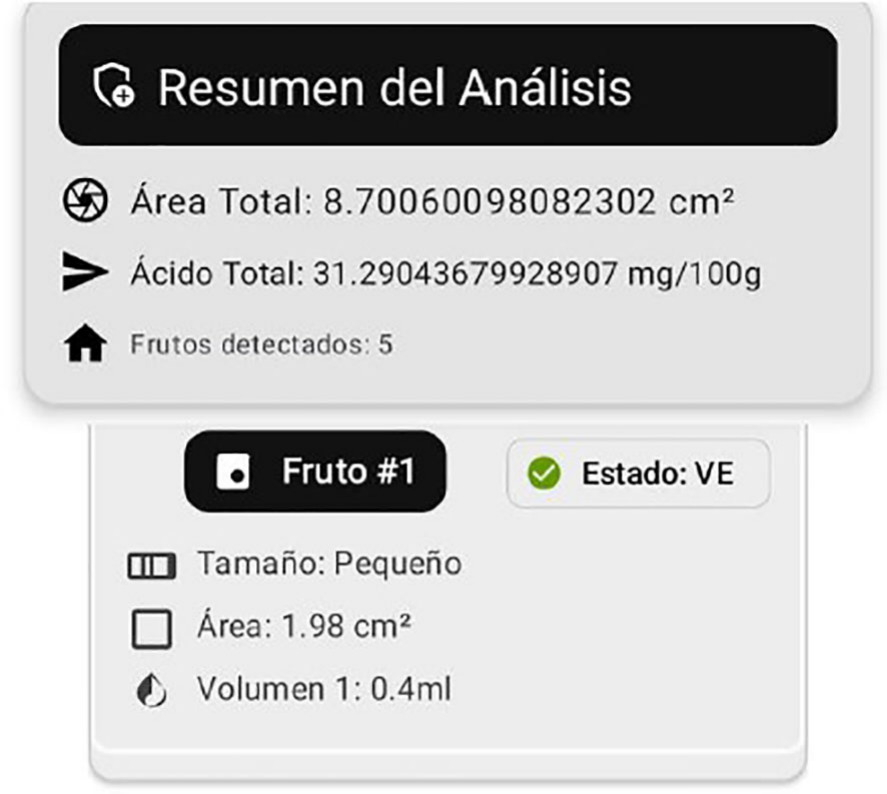
Server response to mobile app: automated fruit analysis and ascorbic acid estimation.

Unlike traditional methods, this system allows rapid field analysis via a mobile app that sends images to a Flask server for automated processing and reporting (**Figure 12**).

The system summarizes results per image, reporting total fruit area, ascorbic acid content, and fruit count, as well as per-fruit size, area, estimated pulp, and ripeness. This enables precise, real-time quality assessment for producers, and supports future machine learning integration show result in **Table 3**.

### 4.5 Limitations and future work

While the proposed system demonstrates high precision for camu-camu, further research is required to validate its transferability to other crops and to improve pulp volume estimation. Future integration of additional sensors (such as NIR or hyperspectral imaging) and advanced artificial intelligence models is expected to enhance the accuracy of biochemical quantification and pulp yield estimation.

#### 4.5.1 Impact of camera distance

A key limiting factor in the accuracy of the system is the camera-to-fruit distance during image capture. Since the estimation of real-world area and subsequent ascorbic acid content relies on a fixed pixel-to-centimeter conversion, any deviation from the recommended 30-cm distance introduces proportional scaling errors. Field tests confirmed that even small changes in distance can result in noticeable errors in area estimation, which directly impact pulp volume and vitamin C quantification.

To minimize this source of error, all images in this study were acquired at a controlled distance using a reference guide. Future work will address automated distance calibration or depth sensing to improve flexibility and robustness under diverse field conditions.

## 5 Discussion

To assess the final precision of the system, **Table 3** compares the levels of ascorbic acid measured in the laboratory with those estimated by our proposed method.

The Mean Absolute Percentage Error (MAPE) Equation (1) was calculated to quantify the difference between the system predictions and laboratory results:

(1) $$MAPE=\frac{1}{n}\sum_{i=1}^{n}\left|\frac{y_{i}-{\hat{y}}_{i}}{y_{i}}\right|\times 100,$$

where $y_{i}$ are the results by laboratory values, ${\hat{y}}_{i}$ are the predicted values, and n is the total number of observations. Since green fruits are not used in practical commercialization, they were excluded from the error analysis. **Table 7** summarizes the MAPE values for the remaining ripeness stages. The errors were slightly higher for the Pinton Green stage due to its color variability, while lower errors were achieved for Ripe and Ripe Pinton stages, making the system highly suitable for practical applications.

**Table 7:** MAPE values for different ripeness stages.

Comparative errors	
Ripeness stage	MAPE
Pinton Green	11.95%
Ripe Pinton	3.8%
Ripe	5.56%
MAPE	**7.1%**

The bold values indicate the MAPE (Mean Absolute Percentage Error) calculated according to Equation (1).

**Figure 13:**
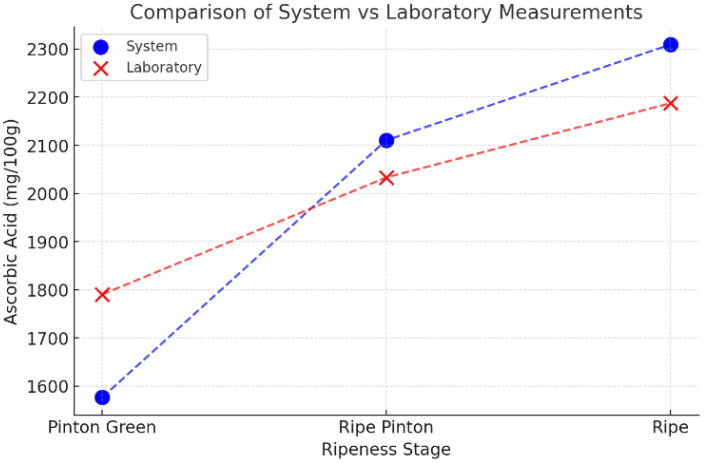
Scatter plot of laboratory vs. predicted ascorbic acid values across ripening stages. The regression line indicates strong correlation (R^2^ = 0.93).

The average estimation error (mean MAPE) across all evaluated ripeness stages was 7.1%, confirming the accuracy and practical viability of the proposed approach for ascorbic acid quantification. **Figure 13** illustrates the strong correlation between measured and predicted values (R^2^ = 0.93), further validating the system’s predictive reliability.

Error Analysis and Sources of Uncertainty: Several factors may explain the observed error. Variations in lighting during image capture, minor inconsistencies in fruit segmentation, and natural variability in fruit morphology not fully captured by the regression model all contribute to uncertainty. Experimental variability in laboratory titration and occasional mislabeling of ripening stages may also have affected accuracy.

The exclusion of green-stage fruits from error analysis follows commercial practices, since these fruits are not typically harvested or traded due to low pulp content. Nevertheless, the system is technically capable of processing green samples, which could be relevant in physiological studies or breeding programs. Future datasets may incorporate these stages to broaden applicability beyond commercial contexts.

Compared to prior works applying CNNs or hyperspectral imaging for nutrient estimation in fruits—where typical errors range from 8–12% (Wang et al., 2021; Zhang et al., 2023)—our approach achieves a lower mean error (7.1%) using only RGB imaging. This highlights the effectiveness of combining transformer-based architectures with regression calibration for vitamin C estimation, while relying on simpler, cost-effective imaging setups.

Inference Speed: To support the claim of real-time processing, we measured the inference speed of both detection models on an NVIDIA RTX 5080 GPU (batch size = 128). RT-DETR achieved an average inference time of 20 ms per image, while YOLOv8 and YOLOX processed images in 13 ms on average. Although YOLOv8 exhibited slightly faster processing speed, RT-DETR delivered superior detection accuracy and recall, which justifies its integration into our pipeline. Both models are suitable for near-real-time field deployment.

**Figure 14:**
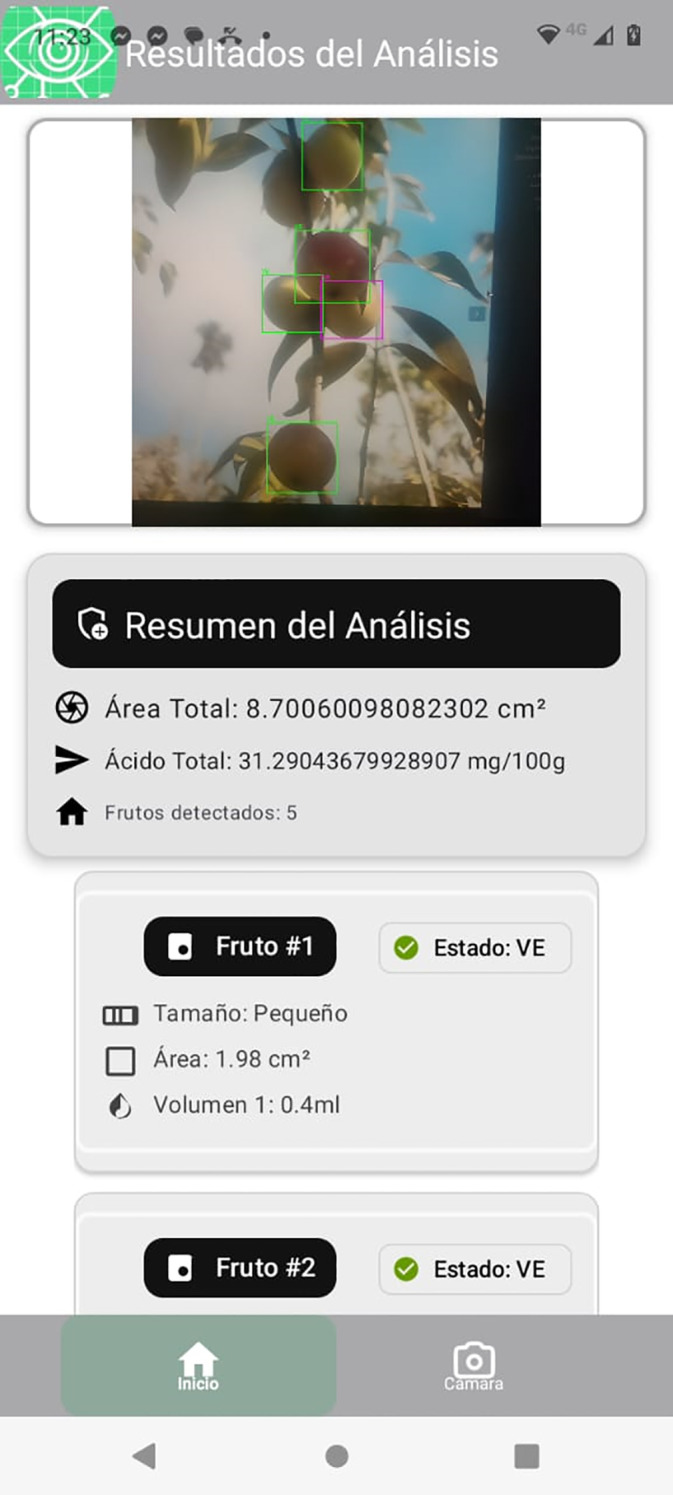
Field implementation of the automated ascorbic acid detection system.

Field Validation and Practical Relevance: Our approach demonstrated robust performance not only under controlled conditions but also in field environments. **Figure 14** illustrates the system’s application during real harvest conditions, where images were captured using a mobile phone and uploaded to a server for processing. The method remained effective as long as the camera-to-fruit distance was maintained at 30 cm. This reliance on mobile phones—technology already familiar to most farmers—positions the system as a scalable tool for smallholder agriculture, where access to laboratory facilities is limited but smartphone penetration is high.

On Model Generalization and Distance Constraints: To ensure practicality and ease of use, we avoided training separate models for different camera distances or devices. Instead, we standardized acquisition with a fixed 30 cm distance, maintained through a simple physical reference. This ensures consistent scale conversion and avoids the need for additional calibration or specialized hardware. While training separate models for variable distances could increase flexibility, it would also add complexity and limit accessibility for non-specialists. As future work, we propose integrating automated distance estimation using visual markers, depth cameras, or machine learning, enabling greater robustness without requiring multiple models.

Original Contribution: To our knowledge, this is the first study to apply transformer-based models to estimate ascorbic acid levels in camu-camu directly from RGB images. By bridging the gap between laboratory precision and field-ready automation, our work contributes a novel, accessible, and cost-effective approach to precision agriculture, with potential for large-scale adoption in fruit quality assessment.

## 6 Conclusions

This study introduces an automated approach to measure ascorbic acid levels in camu-camu fruits using computer vision. By combining high-resolution imaging with advanced models—RT-DETR for detection and Vision Transformer (ViT) for classification—our method provides a practical alternative to laboratory based techniques.

The RT-DETR achieved superior detection performance (precision 0.970, recall 0.976) compared to YOLOv8 (precision 0.946, recall 0.913). Likewise, ViT accurately classified fruits into ripening stages with an accuracy of 0.970, outperforming VGG16 (accuracy 0.946). This confirms the strength of transformer based architectures for agricultural applications.

The proposed system achieved an overall MAPE of 7.1%, demonstrating reliable accuracy for estimating ascorbic acid levels from fruit images. The exclusion of green-stage fruits—due to their minimal pulp content—ensures the estimates are consistent with commercial practices. The creation of a curated image database and the integration of cutting-edge machine learning models represent significant contributions to precision agriculture.

Despite the strong performance, variations in lighting, fruit orientation, or image quality might affect the model’s consistency. Future work could include adaptive lighting normalization methods, expanded training datasets, and exploration of real-time super-resolution techniques to enhance image clarity and analysis precision further.

In conclusion, this method offers an accessible, cost-effective, and scalable solution for assessing camu-camu quality. Beyond its immediate application, it has the potential to transform quality control practices in the fruit industry, bridging the gap between laboratory analysis and field-ready tools. Future work will focus on embedding the system into mobile platforms to further enhance usability and reach.

## Data Availability

The dataset generated and analyzed for this study is available in Roboflow at the following link: https://universe.roboflow.com/universidad-8wt9d/frutas-gl38a/dataset/1. The raw data supporting the conclusions of this article are available from the corresponding author upon reasonable request.
